# Diversity and Distribution of *N*-Acylhomoserine Lactone (AHL)-Degrading Activity and AHL-Lactonase (AiiM) in Genus *Microbacterium*

**DOI:** 10.1264/jsme2.ME11341

**Published:** 2012-03-23

**Authors:** Wen-Zhao Wang, Tomohiro Morohoshi, Nobutaka Someya, Tsukasa Ikeda

**Affiliations:** 1 Department of Material and Environmental Chemistry, Graduate School of Engineering, Utsunomiya University, 7–1–2 Yoto, Utsunomiya, Tochigi 321–8585, Japan; 2 Hokkaido Agricultural Research Center (HARC), National Agriculture and Food Research Organization (NARO), 9–4 Shinsei-minami, Memuro-cho, Kasai-gun, Hokkaido 082–0081, Japan

**Keywords:** quorum sensing, *N*-acylhomoserine lactone, quorum quenching, AHL lactonase, *Microbacterium* sp

## Abstract

*N*-Acylhomoserine lactone (AHL)-degrading enzyme, AiiM, was identified from the potato leaf-associated *Microbacterium testaceum* StLB037. In this study, we cloned eight *aiiM* gene homologues from other AHL-degrading *Microbacterium* strains. The similarity of the chromosomal locus of the *aiiM* gene is associated with the phylogenetic classification based on 16S rRNA. Degenerate PCR revealed that the *aiiM* gene was only conserved in AHL-degrading *Microbacterium* strains, but not in fifteen *Microbacterium* type strains or two *Microbacterium* isolates from other plants. These results suggested that the high level of AHL-degrading activity in *Microbacterium* strains was caused by the *aiiM* gene encoded on their chromosome.

Quorum sensing is a population density-dependent regulation mechanism used by bacteria to regulate gene expression. In many Gram-negative bacteria, several types of *N*-acyl-l-homoserine lactones (AHLs) have been identified to be signal compounds involved in quorum sensing (2, 6). When AHL concentration increases and reaches a threshold due to the accumulation of AHL derived from each bacterial cell, AHL receptor proteins belonging to the LuxR protein family bind AHL and regulate the expression of many genes responsible for bioluminescence, pigment production or antibiotics production (2, 6). In particular, many Gram-negative plant pathogens control the expression of virulence factors by their quorum-sensing systems (13). In general, AHL-negative mutants show defects in their pathogenicity, so it is expected to inhibit the expression of virulence and infection of host cells by disrupting quorum-sensing signals. Many AHL-degrading genes have been cloned and characterized from various bacteria (12). AHL lactonase catalyzes AHL ring opening by hydrolyzing lactones and AHL acylase hydrolyzes the amide bond of AHLs (12). AHL-degrading genes have been utilized to prevent diseases by bacterial plant pathogens. The expression of AHL-lactonase in *Pectobacterium carotovorum* subsp. *carotovorum* significantly attenuates pathogenicity on some crops (3). Transgenic plants expressing AHL-lactonase exhibited significantly enhanced resistance to infection by *P. carotovorum* subsp. *carotovorum*(4).

In our previous studies, we isolated AHL-producing and -degrading bacteria from the leaf and root surface of potato (7, 10, 11). From the potato leaf, we isolated genus *Microbacterium* as the major AHL-degrading bacteria (7). We also cloned a novel AHL-degrading gene (*aiiM*) from *M. testaceum* StLB037 (8, 14). AiiM has AHL-lactonase activity and belongs to the α/β hydrolase fold family (8, 14). In this study, we investigated the diversity and distribution of the *aiiM* gene in the various *Microbacterium* strains.

Based on the results of phylogenetic analysis of the 16S rRNA genes, we divided nine potato-associated and AHL-degrading *Microbacterium* strains into 3 groups (Fig. 1). We previously cloned the *aiiM* gene from StLB037, which belongs to group I. For cloning novel AHL-degrading genes from other groups, we selected two other strains, StLB018 from group II and StLB069 from group III. For cloning the AHL-degrading gene from StLB018 and StLB069, the *E. coli* DH5α/pLux28 reporter system was used as described previously (14). As a result, *E. coli* DH5α harboring pST18-1 clone from StLB018 or pST69-1 clone from StLB069 showed obvious AHL-degrading activity. When the inserted chromosomal fragments were sequenced, it was revealed that both pST18-1 and pST69-1 clones contained *aiiM* homologous ORFs. The DNA sequence of *aiiM* from StLB037 exhibited 88 and 77% identity with the *aiiM* from StLB018 and StLB069, respectively (Fig. 2). To confirm whether other AHL-degrading *Microbacterium* strains also conserve *aiiM* homologues, degenerate PCR primers, AiiMDG-F (5′-ATG ATC CTC GCC CAC GAC-3′) and AiiMDG-R (5′-CGC CAT CTG CGC GAA CAT-3′), were designed based on the most conserved nucleic acid sequences of the *aiiM* genes from StLB018, StLB037, and StLB069 (Fig. 2). As a result of PCR amplification, 438 bp DNA fragments were successfully amplified from all AHL-degrading *Microbacterium* strains and all PCR fragments contained partial *aiiM* sequences. To amplify the upstream and downstream regions of the partial *aiiM* genes from *Microbacterium* strains, inverse PCR was carried out. As a result of sequencing, complete sequences of all *aiiM* genes consisted of 756 nucleotides. In addition, *E. coli* DH5α harboring *aiiM* genes from all *Microbacterium* strains showed AHL-degrading activities against various AHLs (data not shown). These results demonstrated that the *aiiM* gene is widely conserved in AHL-degrading *Microbacterium* strains and has AHL-degrading activity. The nucleotide sequences of the *aiiM* gene and amino acid sequences of AiiM showed more than 76% sequence similarity, and the similarity of the sequence of the *aiiM* gene and AiiM was associated with the phylogenetic classification based on 16S rRNA in *Microbacterium* strains (Fig. 3). To indentify the chromosomal locus of the *aiiM* gene in *Microbacterium* strains, we compared the upstream and downstream regions of *aiiM* genes (Fig. 4). Glyoxalase/ bleomycin resistance gene homologues were located upstream of the *aiiM* gene in group I and II strains. On the other hand, this gene homologue was downstream of the *aiiM* gene in group III strains. In group I strains except for StLB106, tetratricopeptide repeat sequences were located downstream of the *aiiM* gene, and putative membrane protein genes were upstream of the *aiiM* gene in group III strains. These results demonstrated that the similarity of the chromosomal locus of the *aiiM* gene is also associated with the phylogenetic classification based on 16S rRNA in AHL-degrading *Microbacterium* strains.

To examine the presence of AHL-degrading activity in various *Microbacterium* strains, fifteen type strains and two laboratory stock strains were used for the AHL-degrading assay. Fifteen type strains were obtained from the American Type Culture Collection (ATCC) or NITE Biological Resource Center (NBRC). Strain PcRB024 and BrnRB015 were isolated from the root of rapeseed and scarlet runner bean, respectively. *Microbacterium* strains were cultivated in tryptic soy broth (TSB; Nippon Becton Dickinson, Tokyo, Japan) containing 10 μM *N*-hexanoyl-l-homoserine lactone (C6-HSL), *N*-decanoyl-l-homoserine lactone (C10-HSL), *N*-(3-oxohexanoyl)-l-homoserine lactone (3OC6-HSL), or *N*-(3-oxodecanoyl)-l-homoserine lactone (3OC10-HSL) (1). After 6, 12, 24 h, the remaining AHLs were visualized using an AHL-reporter strain, *Chromobacterium violaceum* CV026 (5) or VIR07 (9). The results of the AHL-degrading assay are shown in Table 1. All nine AHL-degrading strains degraded all the tested AHLs within 6 h. In contrast, all type strains and other plant isolates showed no or very weak degrading activity against C6-HSL and 3-oxo-C6-HSL. To confirm the presence of the *aiiM* gene, the internal region of the *aiiM* gene was amplified by degenerate PCR using specific primers designed as above; however, the partial *aiiM* gene fragments were not amplified from either the chromosome of type strains or other plant isolates (Table 1). These results suggested that the high level of AHL-degrading activity in nine *Microbacterium* strains was caused by the *aiiM* gene encoded on their chromosome. Some strains showed high degrading activity against only C10-HSL and 3-oxo-C10-HSL. In a previous study, many AHL acylases showed degrading activity against only AHLs with a long acyl chain (12); therefore, we assumed that the type strains and other plant isolates have putative AHL-acylase.

Many classes of AHL-degrading enzymes have been identified from a wide range of resources, including Gram-positive and -negative bacteria, fungi, plants, animals, humans, and others (12). Although most of these enzymes degrade AHLs in two ways, as AHL-lactonases or AHL-acylases, they showed no significant sequencing identity among different classes (12). Correspondingly, the amino acid sequences of AiiM from AHL-degrading *Microbacterium* strains showed close similarity, but less similarity to a different class of known AHL lactonases (14). *M. testaceum* ATCC 15829 and *Microbacterium* sp. PcRB024 showed a close phylogenetic relationship with the nine AHL-degrading *Microbacterium* strains (Fig. 1), but these two strains did not have significant AHL-degrading activity or conserve the *aiiM* gene in their genomes. These results suggested that the *aiiM* gene was not generally conserved among the genus *Microbacterium*, but was spread among *Microbacterium* strains in a specific ecosystem. However, the transposon insertion site was not found in each sequence around the *aiiM* gene (data not shown); therefore, it was assumed that the *aiiM* gene homologue did not spread to other *Microbacterium* strains by horizontal transmission of the transposon.

In summary, the *aiiM* gene was found in the nine AHL-degrading *Microbacterium* strains but not in any other tested strains by PCR procedures. At this time, the biological and ecological significance of AHL-degrading bacteria and enzymes has not been elucidated. AHL might not be a true substrate for the reported AHL-degrading enzymes; therefore, AHL-degrading *Microbacterium* strains might not exist for the inhibition of quorum sensing in nature. On the other hand, co-inoculation of AHL-degrading strains, StLB018 and StLB037, with *P. carotovorum* subsp. *carotovorum* decreased symptom development in potato soft rot (7). AHL-degrading activity in *Microbacterium* strains might be useful for protecting plants from bacterial infection as symbiotic participants. *Microbacterium* strains are known as endophytic bacteria that reside within plant hosts without causing disease symptoms (15). Also, *Microbacterium* strains, which have no AHL-degrading activity, coexisted with AHL-degrading *Microbacterium* strains on the potato leaf (data not shown). To use potato leaf-associated *Microbacterium* strains as biocontrol agents, it might be important to check for the presence of the *aiiM* gene by PCR using our degenerate primers.

The nucleotide sequences of the *aiiM* gene from *Microbacterium* sp. StLB014, StLB016, StLB018, StLB054, StLB056, StLB069, StLB100 and StLB106 have been deposited in DDBJ/EMBL/GenBank databases under accession nos. AB668537–AB668544, respectively.

## Figures and Tables

**Fig. 1:**
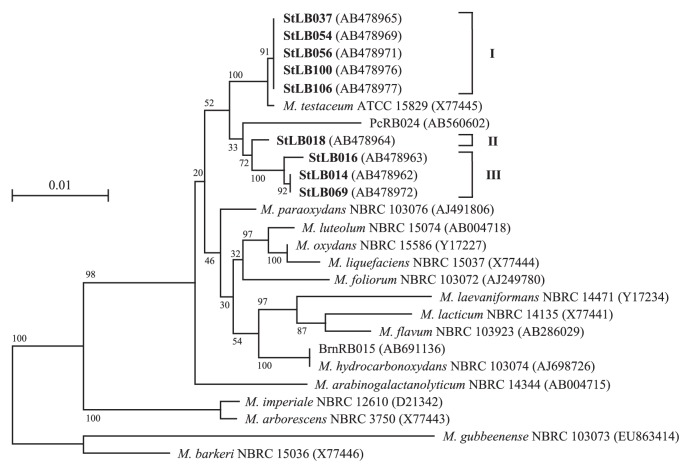
Neighbor-joining tree of the 16S rRNA gene sequences obtained from AHL-degrading *Microbacterium* strains. For phyloge-netic analysis with the 16S rRNA gene, fifteen type strains of the genus *Microbacterium* and two isolates from other plants were used. The potato leaf-associated *Microbacterium* strains are shown in bold. Nine AHL-degrading isolates from potato plants were classed into three groups, I, II, and III. Scale bar represents 0.01 substitutions per nucleotide position. *M. gubbeenense* NBRC 103073 and *M. barkeri* NBRC 15036 were used as the outgroup to root the tree. DDBJ/EMBL/Gen-Bank accession numbers are given in parentheses. Bootstrap values (from 100 replicates) are indicated at the nodes.

**Fig. 2:**
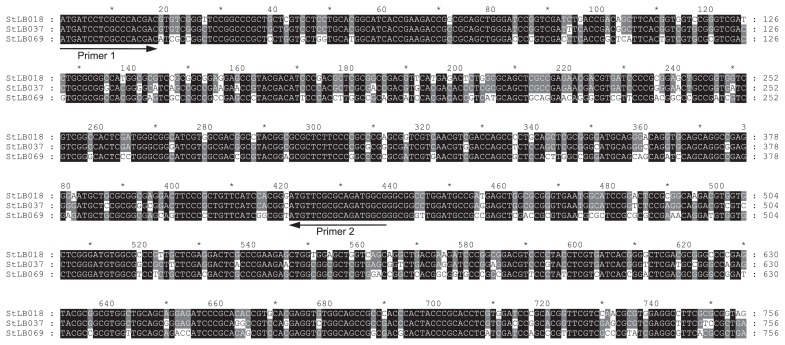
Multiple alignments of DNA sequences of *aiiM* genes from the *Microbacterium* strains, StLB018, StLB037, and StLB069. Sequences were aligned using ClustalW (http://clustalw.ddbj.nig.ac.jp/) and shaded using with the Genedoc program (http://www.psc.edu/biomed/genedoc/). Gray and black shading indicates similar and identical amino acids, respectively. Arrows indicate the primer sites for degenerate PCR.

**Fig. 3:**
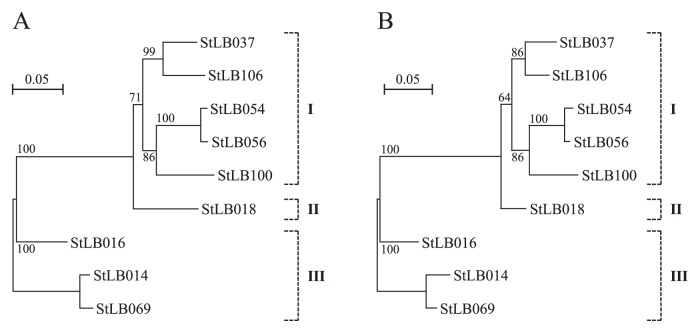
Neighbor-joining trees of the nucleotide sequences of *aiiM* genes (A) and amino acid sequences of AiiM (B) obtained from nine AHL-degrading *Microbacterium* strains. Scale bar represents 0.05 substitutions per nucleotide position. Bootstrap values (from 100 replicates) are indicated at the nodes.

**Fig. 4:**
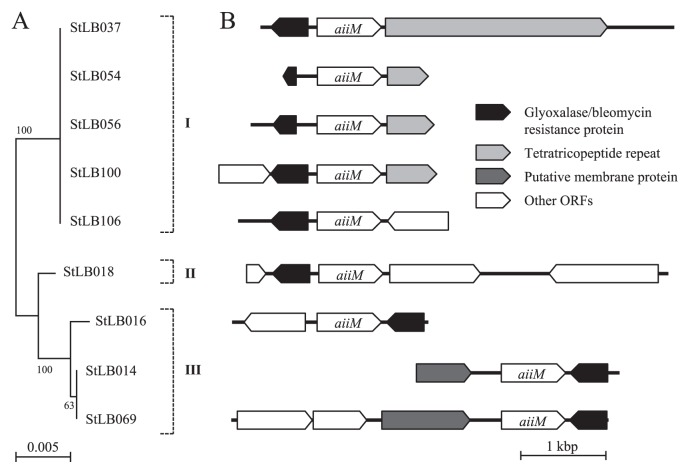
(A) Neighbor-joining trees of the 16S rRNA gene sequences obtained from AHL-degrading *Microbacterium* strains. Scale bar represents 0.005 substitutions per nucleotide position. Bootstrap values (from 100 replicates) are indicated at the nodes. (B) Chromosomal locus of *aiiM* gene in AHL-degrading *Microbacterium* strains. Size, position, and orientation of the identified ORF in each clone are represented by pentagons. Scale bar=1 kbp.

**Table 1:** Identification and characterization of the AHL-degrading bacteria isolated in this study

Strains^a^	Related species	AHL-degrading activity^b^				*aiiM* gene^c^	Source

		C6-HSL	3OC6-HSL	C10-HSL	3OC10-HSL		
StLB014	*Microbacterium* sp.	+++	+++	+++	+++	+	potato
StLB016	*Microbacterium* sp.	+++	+++	+++	+++	+	potato
StLB018	*Microbacterium* sp.	+++	+++	+++	+++	+	potato
StLB037	*Microbacterium testaceum*	+++	+++	+++	+++	+	potato
StLB054	*Microbacterium testaceum*	+++	+++	+++	+++	+	potato
StLB056	*Microbacterium testaceum*	+++	+++	+++	+++	+	potato
StLB069	*Microbacterium* sp.	+++	+++	+++	+++	+	potato
StLB100	*Microbacterium testaceum*	+++	+++	+++	+++	+	potato
StLB106	*Microbacterium testaceum*	+++	+++	+++	+++	+	potato
PcRB024	*Microbacterium* sp.	−	+	+++	++	NA	scarlet runner bean
BrnRB015	*Microbacterium hydrocarbonoxydans*	−	+	−	+	NA	rapeseed
ATCC 15829^T^	*Microbacterium testaceum*	−	+	−	+	NA	Chinese paddy
NBRC 103076^T^	*Microbacterium paraoxydans*	−	+	++	++	NA	blood from a child
NBRC 15586^T^	*Microbacterium oxydans*	−	+	++	++	NA	air
NBRC 14344^T^	*Microbacterium arabinogalactanolyticum*	−	+	+++	+++	NA	soil
NBRC 103074^T^	*Microbacterium hydrocarbonoxydans*	−	+	++	++	NA	soil
NBRC 103923^T^	*Microbacterium flavum*	−	−	+	++	NA	ascidian
NBRC 15074^T^	*Microbacterium luteolum*	−	+	++	++	NA	soil
NBRC 103072^T^	*Microbacterium foliorum*	−	+	+	++	NA	phyllosphere of grasses
NBRC 103073^T^	*Microbacterium gubbeenense*	−	−	−	−	NA	smear-ripened cheese
NBRC 14135^T^	*Microbacterium lacticum*	−	−	+	+	NA	unknown
NBRC 15037^T^	*Microbacterium liquefaciens*	−	+	+	+	NA	milk
NBRC 12610^T^	*Microbacterium imperiale*	−	+	+	+	NA	imperial moth
NBRC 15036^T^	*Microbacterium barkeri*	−	−	−	−	NA	sewage
NBRC 14471^T^	*Microbacterium laevaniformans*	−	−	−	−	NA	activated sludge
NBRC 3750^T^	*Microbacterium arborescens*	−	+	+	−	NA	unknown

a T, type strain.

b −, no degrading activity observed; +, low degrading activity (completely degraded 10 μm AHL within 24 h); ++, intermediate degrading activity (completely degraded 10 μm AHL within 12 h); +++, high degrading activity (completely degraded 10 μm AHL within 6 h).

c NA, not amplified.
